# Relationship among Milk Conductivity, Production Traits, and Somatic Cell Score in the Italian Mediterranean Buffalo

**DOI:** 10.3390/ani12172225

**Published:** 2022-08-29

**Authors:** Roberta Matera, Gabriele Di Vuolo, Alessio Cotticelli, Angela Salzano, Gianluca Neglia, Roberta Cimmino, Danila D’Angelo, Stefano Biffani

**Affiliations:** 1 Dipartimento di Medicina Veterinaria e Produzioni Animali, Università degli Studi di Napoli Federico II, 80131 Naples, Italy; 2 Istituto Zooprofilattico Sperimentale del Mezzogiorno, 80055 Portici, Italy; 3 Associazione Nazionale Allevatori Specie Bufalina (ANASB), 81100 Caserta, Italy; 4 Istituto di Biologia e Biotecnologia Agraria (IBBA), Consiglio Nazionale delle Ricerche, 20133 Milan, Italy

**Keywords:** electrical conductivity, Mediterranean Buffalo, udder health

## Abstract

**Simple Summary:**

Mastitis can be considered one of the costliest diseases of the dairy Mediterranean Buffalo. Early detection of the disease is thus of great importance to farmers, to reduce or prevent production losses. The measurement of electrical conductivity (EC) of milk is a relatively simple and inexpensive technique, and it has been studied as a routine method for the diagnosis of mastitis in dairy farm. Limited information is currently available on the relationships among EC, production traits and somatic cells count in Italian Mediterranean Buffalo. Hence, the aim of this study was to investigate those correlations using data collected at a commercial Italian buffalo farm. We can conclude that in buffalo, as in other species, there is a strong relationship between EC and somatic cells. Furthermore, we observed that, if the objective is to have an additional and informative parameter for early detection of mastitis, frequent EC recording, possibly performed over a longer period of time, is more effective than recording EC only a few times. Even if our results are encouraging, further studies are needed, in order to validate them, especially if the objective is the development of udder disease prediction models.

**Abstract:**

The measurement of milk electrical conductivity (EC) is a relatively simple and inexpensive technique that has been evaluated as a routine method for the diagnosis of mastitis in dairy farms. The aim of this study was to obtain further knowledge on relationships between EC, production traits and somatic cell count (SCC) in Italian Mediterranean Buffalo. The original dataset included 5411 records collected from 808 buffalo cows. Two mixed models were used to evaluate both the effect of EC on MY, PP and FP and EC at test-day, and the effect of EC on somatic cell score (SCS) by using five different parameters (EC_param), namely: EC collected at the official milk recording test day (EC_day_0_), EC collected 3 days before official milk recording (EC_day_3_), and three statistics calculated from EC collected 1, 3 and 5 days before each test-day, respectively. All effects included in the model were significant for all traits, with the only exception of the effect of EC nested within parity for FP. The relationship between EC and SCS was always positive, but of different magnitude according to the parity. The regression of EC on SCS at test-day using different EC parameters was always significant except when the regression parameter was the slope obtained from a linear regression of EC collected over the 5-day period. Moreover, in order to evaluate how well the different models fit the data, three parameters were used: the Average Information Criteria (AIC), the marginal R_2_ and the conditional R_2_. According to AIC and to both the Marginal and Conditional R_2_, the best results were obtained when the regression parameter was the mean EC estimated over the 5-day period.

## 1. Introduction

Buffalo breeding is an important economic asset in Italy, especially in the southern regions. Italy holds about 0.01% of the world buffalo population and the number of animals has increased significantly in recent years) [1]. The reasons for the growing interest in buffalo farming are to be found in the popularity of traditional dairy products, which are obtained from the milk of these animals, and especially in the production of “Mozzarella di Bufala Campana”, a typical cheese characterized by a protected designation of origin (PDO) label [2,3]. Indeed, milk quality is a crucial issue for the Italian buffalo dairy industry, having a direct impact on the technological characteristics of milk itself [4]. It is also well known that milk quality is strictly related to mammary gland health [5,6,7]. Indeed, mastitis is one of the most expensive diseases in the dairy industry [2]. Mastitic milk is characterized by a high number of somatic cells and by changes in its composition, which affects coagulation capacity with consequent production of low-quality cheeses. Diagnosis of clinical mastitis is commonly based on local and systemic reactions or on milk changes. The diagnosis of subclinical mastitis, on the contrary, is more difficult because both milk and udder do not show evidence of abnormality, although, also in this case, there is a high number of somatic cells [8].

According to the guidelines of the International Dairy Federation, the diagnosis of mastitis is mainly based on somatic cell count (SCC) and bacteriological culture of milk, although alternative indicators from milk can be used, such as lactose [9], differential somatic cell count [10,11], L-Lactate dehydrogenase [12] and Electrical Conductivity (EC). The latter is determined by the total concentration of cations and anions in milk, whose values change during an inflammatory process of mammary gland. In fact, during mastitis the blood–milk barrier is damaged and consequently the tight junctions between secretory cells becomes leaky. This promotes the movement of extracellular fluid components, including sodium (Na) and chloride (Cl), that mix with milk and increase the Na and Cl concentrations [13] with a concurrent decrease in milk K concentration [14]. For this reason, EC is considered a reliable indicator of early mastitis diagnosis in dairy cattle [12,15]. The reliability of EC as an early indicator of mastitis has been demonstrated in several species. Milner et al. [16] observed changes in EC after direct infusion of *Staphylococcus aureus* or *Streptococcus uberis* into the mammary gland in lactating Friesian-Holstein cows. Similarly, the intramammary infection of bacteria in Murciano-Granadina goats caused an increase of both SCC and EC [17]. Recently, it has been proposed that EC alone was not sufficient to achieve the desired sensitivity and specificity targets and that improvements can be obtained by using other information (e.g., milk yield, milk flow, number of incomplete milking), that may increase accuracy of detection and ability to determine early onset of mastitis [18]. Other authors [19] suggested the combination of EC, milk production rate and average milk flow rate as a tool potentially useful for an early detection of mastitis.

Although buffaloes are traditionally considered less susceptible to mastitis than cattle [2], some studies have reported high prevalence of subclinical intramammary infections [20,21]. Therefore, the pathology is underestimated in this species, causing both health problems to the animals and noticeable economic loss for the farmers. Because of this, the development of techniques for early detection of mastitis in the farm would allow a rapid and early management of the disease, in order to decrease the negative effect on milk quality and therefore on the livestock market economy.

Limited information is currently available on the relationships among EC, production traits and SCC in Italian Mediterranean Buffalo. In particular, a gap of knowledge is present on the value of EC as a predictive indicator of SCC increase and mastitis in this species. Hence, the aims of this study were i) to investigate the correlations among EC, SCC and production traits in buffalo species and ii) to estimate the predictive value of EC to diagnose SCC increase in buffalo species, by using data collected at a commercial Italian buffalo farm.

## 2. Materials and Methods

### 2.1. Animals and Data

Animal welfare approval was not needed for this study because data came from pre-existing databases. All data were recorded at a commercial buffalo farm located in Cerignola, Foggia (41.2656° N, 15.8936° E) in the southeastern region of Italy, where about 1000 adult heads were present. The original dataset included 5411 records collected from all the lactating buffalo cows (n = 808) from January 2018 to November 2018 and from January 2019 to February 2019. Information related to each individual record included: animal ID, birth date, calving date, parity order, stage of lactation, milk yield (MY), fat percentage (FP), protein percentage (PP), SCC and milk EC. Monthly milk yields and SCC were provided by the official milk recording service of the Italian Breeders Association. Due to their non-normal distribution, SCC were log-transformed into somatic cell score (SCS) using the formula proposed by [22]:

(1) $$\mathrm{SCS}=log_{2}\left(SCC/100,000\right)+3.$$

Buffaloes were housed in free stall barns with a concrete floor. An availability of space of 15 m^2^/head and 80 cm front manger were guaranteed throughout the study. Straw was used for bedding and it was renewed every two days. Animals were subjected to two daily milkings in herring-bone milking parlors equipped with an Afimilk^®^ milk analysis system (AFI-MILK^®^, TDM, San Paolo, Italy). Before milking, the animals had free access to water and, in any case, the waiting time before milking was less than 75 min. Milk EC was recorded directly from the herd milking unit and was available at the official milk recording date ($EC_{0}$) and one ($EC_{1}$), three ($EC_{3}$) and five ($EC_{5}$) days before, respectively.

The stage of lactation (SOL) was evaluated by considering a 30-day in milk (DIM) interval, resulting in 11 classes (class 1 from 1 to 30 DIM; class 2 from 31 to 60 DIM; class 3 from 61 to 90; class 4 from 91 to 120; class 5 from 121 to 150; class 6 from 151 to 180; class 7 from 181 to 210; class 8 from 211 to 240; class 9 from 241 to 270; class 10 from 271 to 300; class 11 from 301 to 330). Parity was grouped into 5 classes, where 5+ parity included animals that were in their 5th or greater parity (maximum parity = 8). Months and year of milk recording were grouped into 11 classes (class 1 January 2018; class 2 February 2018; class 3 March 2018; class 4 April 2018; class 5 June 2018; class 6 July 2018; class 7 August 2018; class 8 September 2018; class 9 October 2018; class 10 November 2018; class 11 January and February 2019). A minimum of five records were required per buffalo cow within parity. Additionally, MY, PP and FP outside the range mean ± 3 standard deviations (SD) were excluded. The resulting final data set used for statistical analysis consisted of 4530 test-day records from 741 buffalo cows. Descriptive statistics per each trait by parity class are in Table 1. The number of cows and records per each level of Parity and Stage of Lactation effects were presented in Supplementary Tables S1 and S2.

### 2.2. Statistical Analyses

This study was made of two parts. In the first part, the effect of EC on MY, PP and FP was investigated. Then, in the second part, the focus was on elucidating the effect of EC collected at and before the date of official milk recording on SCS. The effect of EC on MY, FP and PP was analyzed using the following mixed Model (1):

(2) $$y_{ijklmn}=\mu +YM_{i}+SOL_{j}+Par_{k}+{\left(SOL*Par\right)}_{jk}+EC\left(Par_{k}\right)+anim_{l}+\epsilon_{ijklm}$$

where $y_{ijklm}$ is the dependent variable test-day MY, FP or PP, μ is the overall mean, $YM_{i}$ is the fixed effect for year-month of milk sampling (*i* = 1,…, 11); ${\left(SOL*Par\right)}_{kl}$ is the fixed effect for the interaction between stage of lactation (*k* = 1,…, 11) and parity (*l* = 1,…, 5), EC is a linear regression of EC nested within parity, $anim_{m}$ is the random effect for the buffalo cow and $\epsilon_{ijklmn}$ is the random residual error.

The effect of EC at test-day and the effect of a change in EC during 5 days before test-day on SCS at test-day were investigated fitting the following mixed Model (2):

(3) $$y_{ijklmn}=\mu +YM_{i}+SOL_{j}+Par_{k}+{\left(SOL*Par\right)}_{jk}+EC_{param}\left(Par_{k}\right)+Milk+anim_{m}+\epsilon_{ijklmn}$$

where $y_{ijklmn}$ is the dependent variable test-day SCS, μ is the overall mean, $YM_{i}$ is the fixed effect for year-month of milk sampling (*i* = 1,…, 11), ${\left(SOL*Par\right)}_{kl}$ is the fixed effect for the interaction between stage of lactation (*k* = 1,…, 11) and parity (*l* = 1,…, 5), $EC_{param}$ is a linear regression of an EC parameter nested within parity, MILK is a linear regression on MY, $anim_{m}$ is the random effect for the buffalo cow and $\epsilon_{ijklmn}$ is the random residual error.

The effect of EC on SCS was analyzed using five different parameters ($EC_{param}$), namely: EC collected at the official milk recording test-day ($EC_{day0}$), EC collected 3 days before official milk recording ($EC_{day3}$) and three additional statistics calculated from EC collected 1, 3 and 5 days before each test-day, respectively. Indeed, for each buffalo an EC mean ($EC_{mean}$), an EC standard deviation ($EC_{sd}$) and an EC slope ($EC_{slope}$) were calculated within each 5-day interval before each test-day. The parameter $EC_{slope}$ was estimated from a linear regression of EC on the 5-day period. Then the same Model 2 was repeated five times, including each EC parameter, namely $EC_{day0}$, $EC_{day3}$, $EC_{mean}$, $EC_{sd}$, $EC_{slope}$, respectively.

All the models were fitted using the *nlme* package [23] in R [24]. Akaike information criterion (AIC), marginal and conditional R2 [25,26] were calculated to compare the models and assess which predictors best fitted the data. For all models, hypothesis testing and least-square means for fixed effects were performed and calculated using the *anova* and the *lsmeans* functions from the *stats* [24] and *emmeans* [27] R packages, respectively. Plots were created using package *ggplot*2 [28] implemented in R.

## 3. Results

### 3.1. Descriptive Statistics

**Figure 1:**
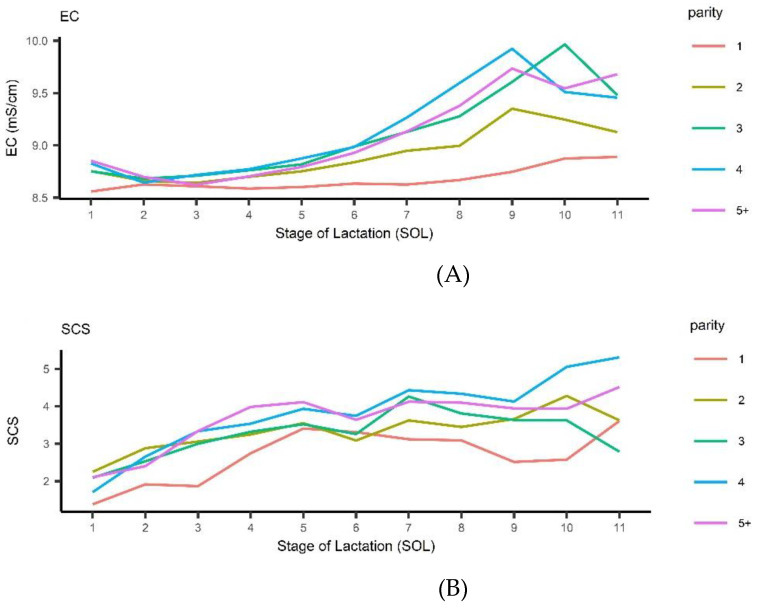
Italian Mediterranean Buffalo: observed trend for electrical conductivity (EC) (**A**) and Somatic Cell Score (SCS) (**B**) across stage of lactation and parity.

The average observed trend for EC and SCS across stage of lactation and parity can be observed in Figure 1.

The EC ranged from a minimum of 8.48±0.37 mS/cm for primiparous within 30 DIM to a maximum of 10.3±2.181 mS/cm for 3rd-parity buffalo cows at 300 dim. Overall, EC increased across lactation, especially for pluriparous buffalo cows from 90 DIM onwards. On the contrary, in primiparous buffalo cows, EC stayed stable below 8.7 across nearly all lactation approaching a nadir at the end (EC = 8.92±0.37). The SCS increased steadily for all parities until 150 DIM, and afterwards the rate of increase changed according to parity.

The phenotypic variability of EC across class of SCS and within parity can be observed in Figure S1. A largest variability was observed when SCS increased, especially in pluriparous buffalo cows. However, high EC values were observed even when SCS was low (<1).

### 3.2. Effect of Milk Conductivity on Milk Yield and Composition

Hypothesis testing for MY, FP and PP from Model 1 is summarized in Table 2.

All effects included in model 1 were significant for all traits, with the only exception of the effect of EC nested within parity for FP. The estimated linear regression coefficients of EC nested within parity for MY and PP are in Table 3.

EC had a significant (unfavorable) effect on MY for all pluriparous buffalo cows. Each increase in EC unit (i.e., the estimate of the linear regression coefficient) reduces milk by a minimum (absolute value) of 0.43847±0.082 kg in 2nd parity to a maximum of 0.62306±0.080 kg in 5th + parity. A similar (unfavorable) relationship was also observed between EC and PP. However, the effect of EC was only significant in 3rd and 5th + parity with a reduction in PP per EC unit increase ranging from −0.02429 to −0.02831%, respectively. The relationship between EC and FP was not significant.

### 3.3. Effect of Milk Conductivity on Somatic Cell Score

The average EC value at official milk recording ($EC_{day0}$), EC collected 3 days before the official milk recording ($EC_{day3}$) as well as its mean ($EC_{mean}$), standard deviation ($EC_{sd}$) and slope ($EC_{slope}$) during the 5-day interval before each test-day were 8.87, 8.85, 8.86, 0.304 and 0.013, respectively.

Hypothesis testing for all models is summarized in Table 4.

The regression of EC on SCS at test-day using different EC parameters was always significant except when the regression parameter was the slope obtained from a linear regression of EC collected in the 5-day period. The estimates of the regression coefficients within parities are in Table 5.

The relationship between EC and SCS was always positive regardless of the EC regression parameter used. The magnitude of the effect varied across parities ranging from a minimum of 0.245 ± 0.074 in 3rd parity, when the regression was on EC collected 3 days before milk recording, to a maximum of 0.953 ± 0.20 in 1st parity, when the regression was on EC standard deviation.

In order to evaluate how well the different models fit the data, three parameters were used: the Average Information Criteria (AIC), the marginal R_2_, which considers only the variance of the fixed effects (i.e., without the random effects), and the conditional R_2_, which takes both the fixed and random effects into account. Results, ordered by the larger conditional R_2_ value (i.e., the best model), are given in Table 6.

According to AIC and to both Marginal and Conditional R_2_, the best results were obtained when the regression parameter was the $EC_{mean}$, followed by the model that fitted a regression on ECday3, on ECday0 and on $EC_{sd}$, respectively.

## 4. Discussion

The electrical conductivity of milk was introduced as an indicator parameter of mastitis and subsequently used in many species, attaining different aims [29,30,31,32]. Indeed, Paudyal et al. [32] carried out a study in Holstein cows and observed that differences in EC and MY characteristic temporal patterns due to particular pathogen groups may provide indications for differentiate groups of mastitis-causing pathogens. Other authors [29,30,31], instead showed that EC measurement in sheep and donkeys is a useful way to identify animals with high SSC levels and with potentially unhealthy mammary glands, thus saving time and money, reducing the cost of other analysis (e.g., bacteriological analyses). Unlike other parameters, such as bacterial culturing-based detection of pathogens, that is still considered a gold standard [33,34] or molecular biological tools, the EC has the advantage of being automatically measured during milking, through electrodes inserted in the milking system. The EC measurement can be converted into a computer-readable signal and therefore this method is easily applicable to on-line automatic udder health monitoring and easily installed in the milking machine [35]. As specified above, an alteration of EC is observed when an alteration of the concentration of some ions is recorded in the milk [13,14]. This feature would allow the breeder to monitor in real-time the udder health of the herd and foresee the onset of problems.

Nowadays, only limited information is available on the usefulness of EC in buffalo species and no previous study investigated EC reference ranges in Mediterranean Buffaloes. The standard range suggested for EC of normal milk in dairy cattle is 4.0–5.5 mS/cm [21]. The mean values of EC for healthy, subclinical and clinical mastitis milk suggested by Norberg et al. [15] are 4.87 mS/cm, 5.37 mS/cm and 6.44 mS/cm, respectively. Overall, we have observed a direct relationship between EC and SCS, which agreed with previous findings in *Bos Taurus*, *Bos Indicus* and *Bubalus bubalis* [36,37,38,39]. However, it is the relationship magnitude depended on parity, being larger for first and fourth parity cows followed by second, third and fifth parity. Observed EC values were higher than those reported by other authors in goats [40,41,42], ewes [43], dairy cattle [38,44,45] or buffaloes [39,46] but similar to those reported in Holstein cows by Paudyal et al. [32].

Parity and SOL were associated with different EC values, as observed in previous studies [32,35,39]. Higher EC values were recorded at both the beginning and the end of lactation, contrary to what was observed in sheep, where the increase was observed only at the beginning of lactation [43]. In particular, in this study [43] a strong increase of EC at the beginning of lactation and only a slight, not significant increase at the end was observed. It is likely that variations in milk composition may have affected these results, since the authors recorded that the milk composition (and particularly the fat concentration) explained high variance in EC. It can be hypothesized that the differences in fat composition may explain our results. In a recent study carried out in cows [45], an EC increase was recorded independently of parity. Interestingly, in our study EC remained almost unchanged throughout lactation only in primiparous buffaloes, whereas a rise was observed in pluriparous counterparts. In particular, for each unit increase in EC, daily milk reduces by a minimum of −0.43 ± 0.082 kg in parity two to a maximum of −0.62 ± 0.080 kg in parity 5+. This relationship between EC trend and parity has also been recorded in other species such as cows [32], sheep [43] and goats [17]. Moreover, the magnitude of the differences due to parity is similar to those previously observed in other studies [32,41]. It cannot be ruled out that this pattern is due to the increased susceptibility of pluriparous to udder damages caused by repeated automatic milking or by some mastitis events [47,48,49].

Regarding milk composition, significant changes in PP were observed in relation to EC with a similar unfavorable effect in 3rd and 5th + parity. This disagrees with previous studies carried out in cattle [50] and sheep [31], where EC was positively associated with milk protein content. According to them, casein would influence the milk conductivity through the insoluble salts that can be linked to the micelles in the colloidal phase. Indeed, casein shows a very low conductance compared to the milk salts. Sometimes, these micelles break down and the salts are released, causing an increase in EC. A possible explanation of the observed unfavorable relationship between EC and PP could be found in the decrease in milk protein content caused by mastitis [39,51]. Indeed, in such a situation a high proteolytic enzymatic activity is observed that eventually damages milk caseins in the mammary gland. It cannot be ruled out that both the higher protein content and the different casein profile recorded in buffalo milk may account for the different results. In this study, no significant relationships were observed between EC and FP, although several authors observed a negative correlation between FP and EC in dairy cows [38], and in goats [40] and ewes [30,43]. According to them, fat globules increase the real distance during ion migration and interfere with the electrodes when measurements of EC are done. Thus, EC is expected to decrease in proportion to FP. Buffalo milk shows about 8% fat content with large variability from 6% to 12% [52] that is more than double compared to cow milk. It is likely that the high fat content and the large variability of fat percentage may have partially affected its relationship with EC. Further studies are needed to investigate this interesting aspect.

Since conductivity is a relatively simple and inexpensive inline detection technique [53], previous researchers have studied EC for the diagnosis of mastitis. Results were controversial and in most of them it was suggested that EC alone cannot be used for this purpose, not being a useful method to determine udder health [18,38]. For this reason, the second aim of the present work was to evaluate the relationship between EC collected 1 ($EC_{day1}$), 3 ($EC_{day3}$) and 5 ($EC_{day5}$) days before official milk recording and SCS at official milk recording ($EC_{day0}$), to eventually develop a prediction model for the detection of the disease. Although no cross-validation models were applied, our results suggest that using data collected at different time points works better than a single data point and that their mean is the best parameter to be included in a possible prediction model. Interestingly, a parameter such as $EC_{slope}$, which is supposed to be more informative being related to a change in EC across the observed period [54], was not significant in relation to SCS. A possible explanation of this result might be related to the reduced observed time period (i.e., 5 days before milk recording) used to estimate $EC_{slope}$. Indeed, fitting a linear regression in such a short period and with only 4 data-points could have shrunk toward zero the estimate of the regression coefficient (i.e., $EC_{slope}$), which eventually was not significant. A similar approach, based on the assessment of electrical conductivity across several days, was also used by Kathun [55]. In this study, a logistic mixed model was used but results showed that for the early detection of mastitis, multiple EC measurements were more informative than a single record. Moreover, Bobbo et al. [56], in a recent study about the prediction of somatic cell count at the subsequent test-day record in the Italian Mediterranean Buffaloes, showed that EC was the 3rd most important source of variation, following SCC-traits recorded at the previous test-day and before other traits as milk production, parity and stage of lactation.

## 5. Conclusions

This is the first study that investigated the electrical conductivity of milk in the Mediterranean Buffalo using data from commercial farm. We can conclude that in buffalo, as in other species, there is a strong relationship between EC and somatic cells. This relationship can be possibly used together with additional parameters for the early detection of mastitis. Indeed, even if EC recording is a simple and low-cost methodology that can also be implemented on large scale, using it as a single predictor may not represent a useful method for determining the health of the udder, since it could be influenced by factors other than mastitis such as parity and/or stage of lactation. However, we observed that if the objective is to have an additional and informative parameter for early detection of mastitis frequent EC recording, possibly performed over a longer period of time, is more effective than recording EC only a few times. This is especially important in buffalo where the incidence of subclinical mastitis is particularly high. Although these are encouraging results, further studies are needed in order to validate them and developing a reliable udder disease prediction model. Finally, our results could establish a reference range or a threshold for EC in the Italian Mediterranean Buffalo. This information may be particularly useful for buffalo breeders for an early recognition of mastitis by the collection of EC that is calculated automatically and in real-time in modern milking machines and could be also inserted in the milk recording procedure.

## Figures and Tables

**Table 1:** Descriptive statistics.

Parity	Milk (kg/d)		Fat %		Protein %		SCS		EC (mS/cm)	
	Mean	Std. Dev.	Mean	Std. Dev.	Mean	Std. Dev.	Mean	Std. Dev.	Mean	Std. Dev.
1	8.96	3.39	9.30	1.93	4.83	0.37	2.73	2.15	8.64	0.50
2	9.97	4.06	8.88	1.81	4.73	0.36	3.24	2.18	8.82	0.84
3	10.84	4.43	8.77	1.78	4.63	0.39	3.26	2.08	8.91	0.96
4	10.42	4.32	8.68	1.79	4.65	0.38	3.58	2.19	9.05	1.07
5+	10.60	4.41	8.49	1.70	4.60	0.38	3.59	2.26	8.99	1.10

**Table 2:** F-value and significance of fixed effects included in the analysis for milk traits and conductivity.

Model ^a^	Trait	YM ^b^	SOL ^c^	Par ^d^	(SOL * Par) ^e^	(EC * Par) ^f^
1	MY (kg/day)	279.3 ***	318.2 ***	32.3 ***	151.3 ***	159.9 ***
1	FP (%/day)	46.1 ***	437.1 ***	1.9 ns	65.9 **	5.7 ns
1	PP (%/day)	323.3 ***	407.6 ***	4.5 ns	88.4 ***	15.3 **

^a^ Statistical significance is given as: ** *p* < 0.01; *** *p* < 0.001; ^b^ year-month of calving; ^c^ stage of lactation; ^d^ parity; ^e^ stage of lactation by parity; ^f^ Milk Conductivity by parity.

**Table 3:** Estimated linear regression coefficients of EC nested within parity for daily milk yield (MY, protein (PP) and fat percentage (FP) in Italian Mediterranean Buffaloes.

Parity	MY ^a^		PP ^b^		FP	
	b	*p* Value	b	*p* Value	b	*p* Value
1	0.0029 ± 0.17	0.99	−0.0014 ± 0.02	0.9436	−0.1308 ± 0.11	0.2452
2	−0.4385 ± 0.08	<0.0001	−0.0101 ± 0.01	0.2906	−0.0415 ± 0.05	0.4393
3	−0.5541 ± 0.09	<0.0001	−0.0283 ± 0.01	0.0091	−0.0425 ± 0.06	0.4784
4	−0.5468 ± 0.09	<0.0001	−0.0089 ± 0.01	0.3948	−0.0051 ± 0.06	0.9282
5+	−0.6231 ± 0.08	<0.0001	−0.0243 ± 0.01	0.0095	−0.0908 ± 0.05	0.0681

^a^ Variation in Daily Milk kg by a 1 mS change in EC; ^b^ Variation in Daily Protein content by 1 a mS in EC.

**Table 4:** F-value and significance of fixed effects included in the analysis for somatic cell score and milk conductivity.

Regression Parameter	YM ^a^	SOL ^b^	Par ^c^	Milk ^d^	(SOL * Par) ^e^	(EC * Par) ^f^
EC at milk recording	77.5 ***	41.6 ***	16.3 ***	91.2 ***	1.2 ns	22.9 ***
EC 3 days before milk recording	77.2 ***	41.2 ***	16.1 ***	90.9 ***	1.1 ns	17.2 ***
EC mean during 5 days before milk recording	77.8 ***	41.5 ***	16.4 ***	91.6 ***	1.2 ns	25.7 ***
EC standard deviation 5 days before milk recording	77.3 ***	40.9 ***	16.1 ***	90.0 ***	1.1 ns	18.9 ***
EC change (slope) 5 days before milk recording	76.64 ***	39.7 ***	14.9 ***	88.6 ***	1.1 ns	0.5 ns

^a^ year-month of calving; ^b^ stage of lactation; ^c^ parity; ^d^ Milk yield; ^e^ stage of lactation by parity; ^f^ Milk Conductivity by parity. *** *p* < 0.001.

**Table 5:** Estimated linear regression coefficients of different EC parameters nested within parity for SCS in Italian Mediterranean Buffaloes.

Parity	EC at Milk Recording		EC 3 Days before Milk Recording		EC Mean During 5 Days before Milk Recording		EC Standard Deviation 5 Days before Milk Recording	
	SCS	*p* Value	SCS	*p* Value	SCS	*p* Value	SCS	*p* Value
1	0.600 ± 0.14	<0.0001	0.26 ± 0.10	0.01115	0.387 ± 0.13	0.0027	0.953 ± 0.21	<0.0001
2	0.391 ± 0.07	<0.0001	0.433 ± 0.07	<0.0001	0.571 ± 0.08	<0.0001	0.612 ± 0.12	<0.0001
3	0.321 ± 0.08	<0.0001	0.245 ± 0.07	0.00099	0.369 ± 0.09	<0.0001	0.476 ± 0.13	0.00021
4	0.408 ± 0.07	<0.0001	0.249 ± 0.06	<0.0001	0.378 ± 0.08	<0.0001	0.635 ± 0.13	<0.0001
5+	0.277 ± 0.06	<0.0001	0.309 ± 0.07	<0.0001	0.409 ± 0.08	<0.0001	0.530 ± 0.14	0.00018

^a^ year-month of calving; ^b^ stage of lactation; ^c^ parity; ^d^ Milk yield; ^e^ stage of lactation by parity; ^f^ Milk Conductivity by parity.

**Table 6:** AIC (Akaike information criterion), marginal and conditional R2 from models used to investigate the relationship between milk conductivity and SCS at test-day.

Regression Parameter	AIC	Marginal	Conditional
EC mean during 5 days before milk recording	18,542	0.247	0.371
EC 3 days before milk recording	18,584	0.240	0.367
EC at milk recording	18,555	0.246	0.365
EC standard deviation during 5 days before milk recording	18,575	0.243	0.364

## Data Availability

The data presented in this study are available on request from the corresponding author.

## Supplementary Materials

The following are available online at https://www.mdpi.com/article/10.3390/ani12172225/s1, Figure S1: Box-Plot Summary of EC data across SCS and within parity; Table S1: Number of cows and records per Parity level, Table S2: Number of records per Stage of lactation level.
