# Supplementary material for: Relationship among Milk Conductivity, Production Traits, and Somatic Cell Score in the Italian Mediterranean Buffalo
Source: Animals (Basel). 2022 Aug 29;12(17):2225. doi: 10.3390/ani12172225 (PMC9455038; doi:10.3390/ani12172225)
Supplement: Supplementary file 1 [file animals-12-02225-s001.zip › animals-1865939-supplementary.pdf]

Supplementary Material

Matera et al.

2022-08-22

Table of Contents

1 Additional Statistics 1

2 Figure S1 2

1 Additional Statistics

The number of cows and records per each level of Parity and Stage of Lactation effects are in table S1 and S2.

Table S1: Number of cows and records per Parity level

parity	Cows[a]	Records
1	132	779
2	229	1,214
3	158	817
4	143	770
5+	159	950

[a]A total of 80 out of 741 cows had 2 parities.

Table S2: Number of records per Stage of lactation level

Stage of lactation[a]	Parity				
1	65	99	75	76	88
2	65	104	82	67	87
3	60	179	108	104	125
4	58	147	109	107	122
5	83	148	106	91	98

Stage of lactation[a]			Parity		
6	104	160	106	102	121
7	116	138	80	82	92
8	100	121	75	79	96
9	74	80	49	41	70
10	40	25	17	17	26
11	14	13	10	4	25

[a]Each stage of lactation class included a 30 days in milk interval.

2 Figure S1

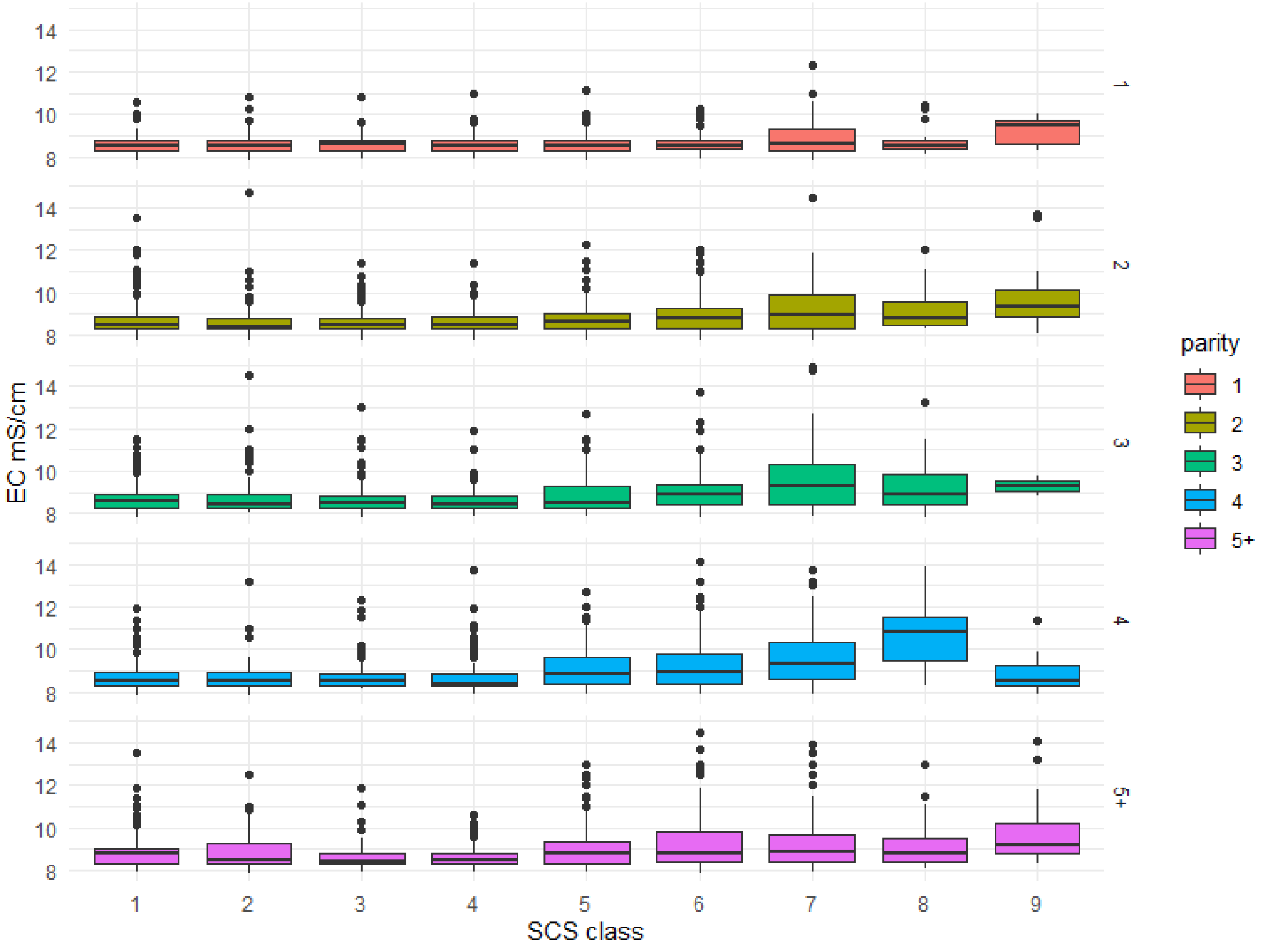

Figure S1: Box-Plot Summary of EC data across SCS and within parity

```
#           #                       p           n           g
##   2

#           #                       p           n           g
##   2

##                          numDF  denDF   F-value  p-value
## (Intercept)                  1   3719  6156.810   <.0001
## dim                         10   3719    39.754   <.0001
## parity                       4   3719    14.898   <.0001
## GC1                         10   3719    76.636   <.0001
## LATTECF                      1   3719    88.634   <.0001
## dim:parity                  40   3719     1.123   0.2752
## parity:estimate     5  3719    0.477  0.7934
```
